# A low-profile high-isolation dual-polarized base station antenna based on AMC

**DOI:** 10.1038/s41598-026-46941-9

**Published:** 2026-04-02

**Authors:** Lijun Zhang, Yongshun Wang, Wenqiang Dang, Xueyun Jiang, Huan Yang

**Affiliations:** 1 School of Electronic and Information Engineering, Lanzhou Jiaotong University, Lanzhou, 730070 China; 2 School of Electronic and Information, Gansu Industry Polytechnic College, Tianshui, 741025 China; 3 Engineering Research Center, Ministry of Education on Integrated Circuit Packaging and Testing, Tianshui, 741001 China; 4 School of Electronic Information and Electrical Engineering, Tianshui Normal University, Tianshui, 741001 China; 5 School of Information Science and Engineering, Lanzhou Bowen College of Science and Technology, Lanzhou, 730101 China; 6 Technology Marketing Center, Huatian Technology (Xi’an) Headquarters Management Co., Ltd., Xi’an, 710018 China

**Keywords:** Dual polarization, Low profile, Artificial magnetic conductor, High isolation, Engineering, Physics

## Abstract

To reduce the deployment cost of base stations and address the shortage of antenna resources, this paper develops a low-profile base station antenna with an Artificial Magnetic Conductor (AMC) structure for Sub-6 GHz. A square cross-dipole structure with chamfered corners is designed, achieving good impedance matching characteristics and significantly improving the port isolation of the antenna. The designed AMC surface structure is used to replace the traditional metal floor of the base station antenna. The suppression of surface waves and phase compensation reduce the profile height of the base station antenna to 0.11$\lambda$. To test its electrical performance, an antenna sample is fabricated. Both simulation and measurement results show that the low-profile base station antenna achieves an impedance bandwidth of 3.1–4.1 GHz, with a port isolation better than 42 dB, a Voltage Standing Wave Ratio less than 1.5, and a gain greater than 8.5 dBi within the operating frequency band, exhibiting stable radiation pattern characteristics.

## Introduction

With the rapid development of wireless communication technology, modern communication systems have put forward higher requirements for the performance of base station antennas. Especially in fields such as 5G communication, Internet of Things (IoT), satellite communication, and military communication, antennas not only need to possess broadband characteristics to support the working requirements of multiple frequency bands and multiple modes but also need to meet the miniaturization design to adapt to the development trends of low profile and integration^1,2^. Dual-polarized antennas improve the efficiency of transceiver duplexing and frequency reuse by transmitting and receiving electromagnetic waves with two mutually orthogonal polarization directions. This technology has been widely applied in mobile communication systems^3–5^. Especially in 5G networks, dual-polarized antennas have become the first choice for base station antennas because they can overcome the multipath fading problem of electromagnetic waves during propagation and improve the reliability of the system. Currently, patch antennas are an ideal choice for dual-polarized and miniaturized antennas, but they usually have a relatively narrow bandwidth^6–9^ or a relatively high profile. Therefore, it is of great significance to achieve a large bandwidth and a low profile for base station antennas.

Dual-polarized dipole antennas have received extensive attention mainly by improving the feeding structure to enhance impedance matching and expand the bandwidth of base station antennas^10–12^. By adopting bending^13–15^ and slotting^16–18^ techniques for dipole antennas, the miniaturization and bandwidth expansion of antennas can be achieved. However, the bending technique will reduce the radiation resistance of the base station antenna, resulting in poor radiation performance and affecting the operating frequency band of the antenna. In contrast, the slotting technique is more conducive to reducing the size while maintaining the radiation characteristics. Based on this idea, the radiation unit is designed in this paper.

However, the profile height of traditional base station antennas needs to be maintained at approximately one-quarter of the wavelength corresponding to their center frequency. This is a limitation of traditional base station antennas in terms of profile height. Although high-profile antennas can achieve better radiation efficiency and bandwidth, they are difficult to meet the requirements of modern devices for a compact structure. While low-profile antennas reduce the size, they often lead to problems such as a narrow bandwidth and a reduced gain. To solve this problem, the AMC structure has been introduced into antenna design. The AMC structure is a periodic electromagnetic material that can achieve the same-phase reflection characteristic within a specific frequency band, thereby effectively improving the performance of the antenna. In recent years, a series of research works have been carried out around the shape and performance improvement of the AMC structure. References^19,20^ used a square AMC structure with a profile height of 0.13$\lambda$. The isolation in reference^19^ is greater than 27 dB, and reference^20^ increased the isolation to greater than 30 dB by changing the feeding structure, the structure of the dipole, and the height of the air layer between the AMC structures. Reference^21^ used an AMC structure with square slots on a square patch. The profile height of the antenna is 0.12$\lambda$, and the isolation is greater than 25 dB. Reference^22^ designed a windmill-shaped AMC structure, effectively reducing the profile height of the antenna to 0.12$\lambda$, with an isolation greater than 28 dB. Reference^23^ proposed a new cross-shaped AMC structure, making the profile height of the antenna 0.15$\lambda$, and the isolation is greater than 27 dB. These results demonstrate that the AMC structure exhibits significant advantages in the design of low-profile base station antennas^24^. More importantly, the high-impedance surface of the AMC can effectively suppress the propagation of surface waves, which is crucial for the dense deployment of Massive MIMO (Multiple-Input Multiple-Output) arrays and the mitigation of inter-element coupling in limited space^25^. Reference^26^ indicates that the adoption of AMC in MIMO antenna systems can effectively enhance the isolation and radiation efficiency. However, how to further boost the isolation and optimize the bandwidth performance of the antenna while minimizing the profile height remains to be investigated in depth.

In this paper, a high-performance antenna with a low profile, large bandwidth, and high isolation based on the AMC structure is designed and implemented. First, a dual-polarized broadband base station antenna element is designed. Through structural optimization, a low-profile base station antenna with high isolation and wide bandwidth is achieved. Then, an AMC structure is designed. Based on the basic principle of the AMC structure, the key parameters affecting its performance are analyzed in depth, and optimization design and simulation verification are carried out. Finally, by optimizing the arrangement of the AMC structure units, a low-profile base station antenna system is constructed, and the performance of the antenna is verified through electromagnetic simulation. The simulation results show that the designed low-profile antenna exhibits good impedance matching characteristics within the frequency band of 3.1–4.1 GHz, with a VSWR less than 1.5, a port isolation better than 42 dB, and a gain greater than 8 dBi within the operating frequency band. The radiation pattern is stable, and the cross-polarization ratio in the main radiation direction is greater than 30 dB. All performance indicators meet the design requirements of the base station antenna. The design scheme proposed in this study provides a new technical approach for the design of low-profile broadband antennas and has important theoretical significance and practical value for promoting their application in modern wireless communication systems.

## Results

### Principle analysis and antenna element design

The size of an antenna is determined by its center frequency. To achieve a broadband characteristic for the antenna, it is necessary to generate additional resonant points for the antenna. In this paper, the broadband characteristic of the antenna is realized by virtue of the multi-mode resonance characteristic. The specific physical principles are described from the following two aspects:

#### Principle of multi-mode resonance and bandwidth enhancement

The bandwidth enhancement of the antenna designed in this paper is based on the principle of multi-mode resonance and mode coupling. Traditional square patch dipoles only support fundamental-mode resonance, which results in a narrow impedance bandwidth that cannot meet the design requirements of broadband base station antennas. To overcome this limitation, this design perturbs the current path on the patch surface by introducing cut corners at the vertices of the patch and cross-slots in the central area: the cut corners are equivalent to introducing asymmetric capacitive loading, which can excite higher-order modes orthogonal to the fundamental mode, while the central cross-slots further extend the current path, introducing a new low-frequency resonant mode.

When the frequencies of multiple resonant points are sufficiently close, their impedance characteristics will couple and superimpose in the frequency domain, connecting the originally independent narrow bandwidths into a continuous wide bandwidth, thus significantly broadening the impedance bandwidth of the antenna. In this design, by optimizing the size of the cut corners and the length and width of the cross-slots, the resonant points are effectively coupled within the 3.1–4.1 GHz frequency band, ultimately achieving a continuous impedance bandwidth with $S_{11} < -10\,\text {dB}$ and a relative bandwidth of 27.8.

#### Mechanism for high isolation achievement

The high isolation ($>42$ dB) between the two ports is mainly attributed to the structural symmetry and decoupling design. First, a pair of crossed dipoles are orthogonally arranged at $90^\circ$ in space, which forms the physical basis for polarization isolation. Second, a unique layout of the “T-shaped” feeding microstrip lines is adopted, where one line achieves interlayer transition via a shorting via, effectively avoiding the direct crossing of the feeding network and reducing the coupling between the feeding lines. Most importantly, the AMC reflective layer plays a crucial role in suppressing surface waves. A traditional PEC ground plane excites intense surface waves that tend to form coupling paths between ports, while the high-impedance characteristic of the AMC can suppress the propagation of surface waves, thus significantly reducing the energy coupled through the ground plane—this constitutes the core physical reason for the isolation being enhanced to above 42 dB.

### Fabrication of the antenna element

**Fig. 1:**
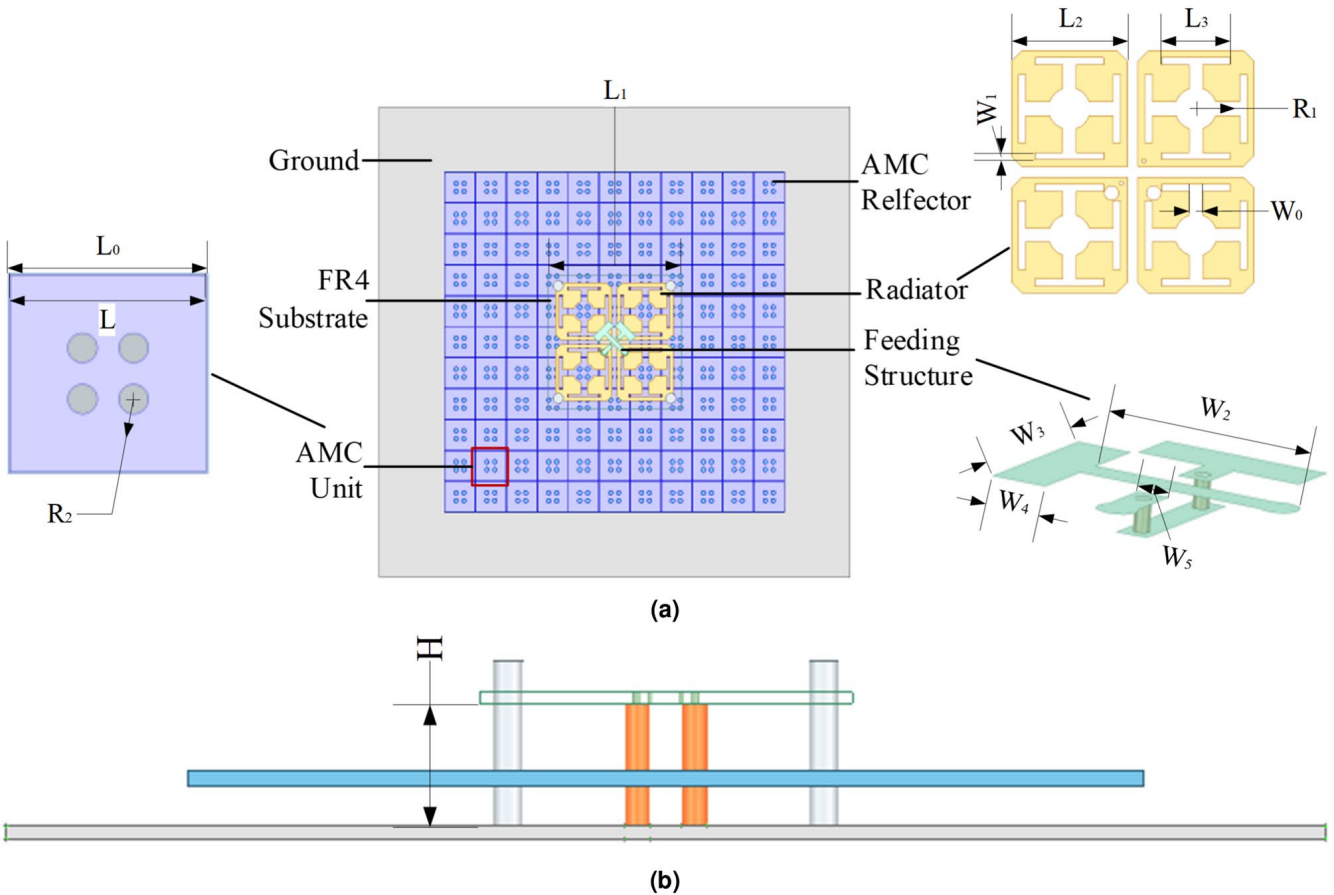
Schematic of the low-profile antenna with AMC reflector, (**a**) top view, (**b**) side view.

A dual-polarized low-profile base station antenna with an AMC structure is fabricated in this paper, and its configuration is shown in Fig. 1. The antenna mainly consists of a radiation unit, a feeding structure, an AMC structure, and plastic columns for support. The radiation unit of the antenna is composed of a pair of cross-dipoles, printed on the bottom of an FR-4 dielectric board with a dielectric constant of 4.4, a loss tangent of 0.02, and a thickness of 0.7 mm. At the same time, chamfering is performed on each patch, and slotting is carried out on the antenna. By changing the current distribution on the surface of the patch, the bandwidth is effectively expanded. To achieve dual polarization and avoid physical crossing of the two feeding lines, a T-shaped microstrip structure is adopted to feed the antenna in this paper. The feeding principle is as follows: the end of the T-shaped microstrip line is extended to form capacitive coupling, which feeds the dipole arms. This non-contact coupling method is more conducive to adjusting the impedance matching compared with direct feeding^27^. The structure consists of two ports, namely Port 1 and Port 2. Port 1 is directly fed from the bottom by a coaxial probe, whose inner conductor is connected to the T-shaped microstrip line on the top layer of the dielectric substrate, and the outer conductor is connected to the radiation patch and the ground plane on the bottom layer. Part of the feeding microstrip line of Port 2 is located on the bottom layer (i.e., the same layer as the radiation patch of Port 1), and is vertically connected to the top layer of the dielectric substrate through a metallized via, then connected to the other arm of the T-shaped microstrip line on the top layer. In this way, the two T-shaped feeding lines are vertically arranged in space, and the in-plane crossing is avoided through the interlayer transition. The AMC unit structures are arranged in a uniform and equidistant manner and placed below the radiation unit and the feeding structure. Finally, a low-profile antenna of 11$\times$11 is obtained. After simulation analysis, the key parameters of the dual-polarized base station antenna are shown in Table 1.

**Table 1:** Antenna structural parameters (unit: mm).

Parameter	Dimension	Parameter	Dimension
L	5.90	$W_0$	1.30
$L_0$	6.00	$W_1$	0.70
$L_1$	23.00	$W_2$	6.20
$L_2$	11.00	$W_3$	1.40
$L_3$	6.50	$W_4$	4.30
H	8.50	$W_5$	0.80
$R_1$	2.00	$R_2$	0.45

The target center frequency of the antenna design is $f_{r0}$ = 3.6 GHz. For the square patch dipole, its arm length $L_1$ is estimated based on the half-wave dipole principle^25^:

1 $$\begin{aligned} L_1 \approx \dfrac{c}{f_{r0}\sqrt{\varepsilon _{\textrm{eff}}}} \end{aligned}$$

Where *c* is the speed of light, and $\epsilon _{eff}$ is the effective permittivity of the FR-4 substrate. From this formula, the dipole arm length of the antenna is calculated to be approximately 25.36 mm, and through optimization and simulation, the value of $L_1$ is determined to be 23 mm.

**Fig. 2:**
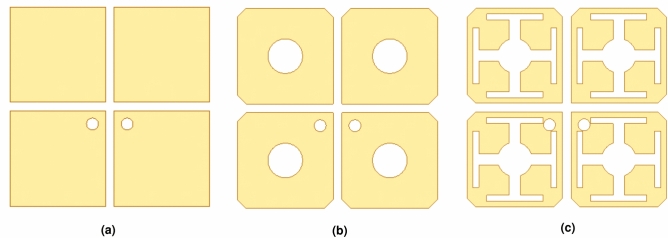
Antenna design evolution: (**a**) Ant.1, (**b**) Ant.2, (**c**) Ant.3.

**Fig. 3:**
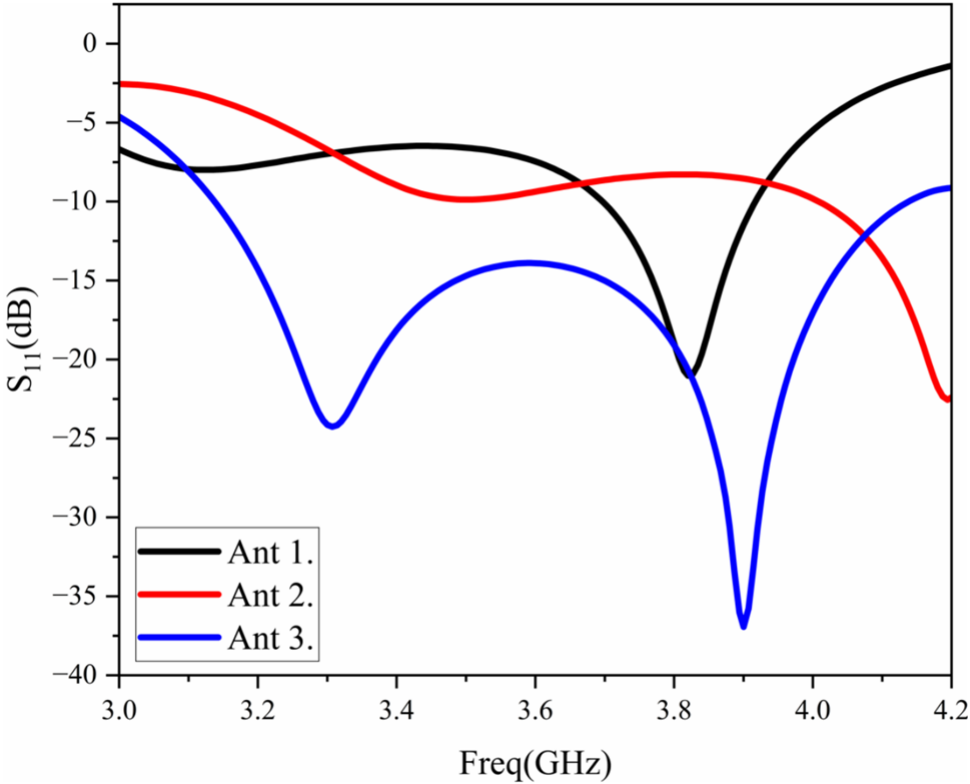
Comparison of S-parameters for the three antennas.

The design evolution of the antenna is shown in Fig. 2, and the simulated S-parameters of the three antennas are shown in Fig. 3.

Antenna 1 (Fig. 2a): consists of basic square patch dipoles. As shown in Fig. 3, its $S_{11}$ curve exhibits only a narrow resonant valley at high frequencies, resulting in a narrow impedance bandwidth that fails to meet the design requirements of base station antennas.

Antenna 2 (Fig. 2b ): cut corners and a central circle are introduced at the vertices of the rectangular patch. The cut corners are equivalent to introducing asymmetric capacitive loading, which disturbs the current path and excites higher-order modes orthogonal to the fundamental mode, thereby introducing a new resonant point $f_{r1}$. The red curve in Fig. 3 shows dual-resonance characteristics, verifying the effectiveness of the cut corners, but the low-frequency bandwidth remains insufficient.

Antenna 3 (Fig. 2c): cross-slots are added at the edges of the patch of Antenna 2. The cross-slots further extend the current path, introducing a new low-frequency resonant point $f_{r2}$ (satisfying $f_{r2}<f_{r0}$) and slightly shifting the original resonant point $f_{r1}$ to higher frequencies. As shown by the blue curve in Fig. 3, by optimizing the slot dimensions, $f_{r2}$ and $f_{r1}$ are coupled in the frequency domain, forming a continuous wide impedance bandwidth, ultimately achieving an operating frequency band of 3.1–4.1 GHz.

### Design and analysis of the AMC structure

In the design of traditional dual-polarized antennas, metal reflector plates are widely used due to their good electrical conductivity. However, due to the inherent 180$^{\circ }$ phase reversal characteristic of the metal surface, a spacing of 0.25$\lambda$ between the antenna and the reflector plate is usually required to meet the impedance matching requirements. Specifically, when an electromagnetic wave propagates backward by 0.25$\lambda$ and reaches the metal surface and is reflected, it undergoes a 180$^{\circ }$ phase reversal. Then, after propagating forward by 0.25$\lambda$, it is in-phase superimposed with the electromagnetic wave radiated by the antenna, thereby optimizing the impedance matching characteristics and radiation performance of the antenna. However, this structure limits the miniaturization design of the antenna. Moreover, when the height of the antenna is reduced, it often leads to problems such as deteriorated impedance matching and decreased radiation efficiency. In recent years, as a new type of electromagnetic material, the AMC structure has received extensive attention due to its unique zero-phase reflection characteristic. Similar to the Perfect Magnetic Conductor (PMC), the AMC structure can achieve the in-phase reflection within a specific frequency band, thus breaking through the 0.25$\lambda$ height limit of traditional antennas. Furthermore, the anti-phase reflection of a perfect electric conductor (PEC) causes coupling with the near field, which partially cancels the radiated energy–this effect is particularly severe in low-profile designs. The in-phase reflection of the AMC constructively superposes with the radiated field, improving the forward gain and radiation efficiency. In contrast, a PEC tends to excite surface waves, which leads to energy diffusion along the ground plane, degrading the sidelobes and increasing port coupling. The high-impedance surface of the AMC can effectively constrain surface waves^28^, which is the key to achieving an extremely high port isolation of $>42$ dB. Most importantly, the reflection phase of the AMC can be flexibly tuned by adjusting the element shape (e.g., the square ring combined with a circle adopted in this paper), dimensions, dielectric layer thickness and other parameters to match the requirements of the radiating element, whereas the reflection characteristics of a PEC are fixed^29^.

The unit structure of the AMC is designed based on the requirements of the target frequency band, and its reflection characteristic bandwidth within the phase range of $- 90^{\circ }$ to + 90$^{\circ }$ is determined through electromagnetic simulation. The unit structures are optimized and arranged according to the array theory, and then the number of units required is determined in combination with specific design specifications to complete the overall design of the reflector plate.

The composite AMC unit designed in this paper aims to achieve a broadband characteristic with $0^{\circ } \pm 90^{\circ }$ reflection phase coverage. Drawing on the concept of composite AMC structures, this design optimizes the reflection phase bandwidth by adjusting the unit’s topology^30^.

**Fig. 4:**
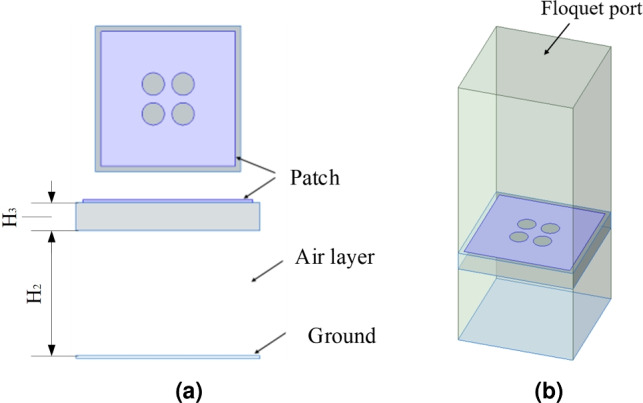
AMC surface configuration: (**a**) unit cell geometry, (**b**) simulation model.

**Fig. 5:**
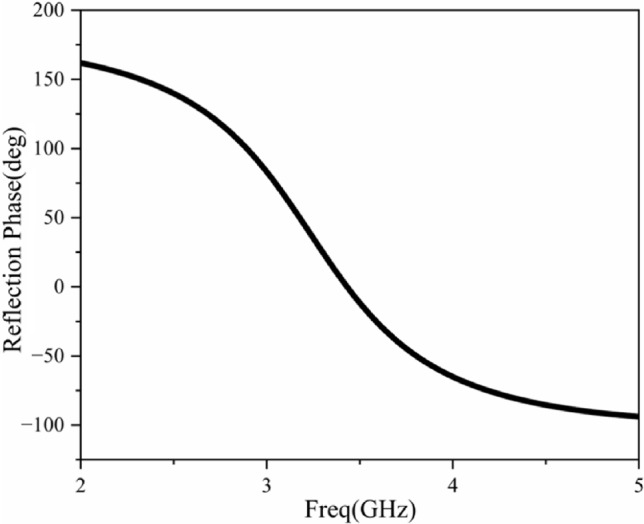
Simulation results of the AMC unit.

The AMC structure is shown in Fig. 4. The unit structure consists of a dielectric board, a metal patch on the upper layer of the dielectric board, a metal floor, and an air layer between the floor and the dielectric layer, where $H_2$ = 3.7 mm and $H_3$ = 1.6 mm. As can be seen from Fig. 4a , there is an air layer between the dielectric board and the floor. This can increase the thickness of the equivalent dielectric board, which is beneficial for achieving a wider same-phase reflection phase. Fig. 4b illustrates the simulation model employing the Floquet port method. All electromagnetic simulations throughout this study were carried out using Ansys High Frequency Structure Simulator (HFSS) 2024 R1, which is available at the official website: https://www.ansys.com/products/electronics/ansys-hfss. Two pairs of master-slave boundary conditions are set on the side of the unit structure, a Floquet port is set directly above it, and the surface patch is set as the reference plane using the deem operation. In this way, the reflection phase curve of the periodic structure can be obtained, as shown in Fig. 5. The reflection phase of the designed AMC is 2.5–4.5 GHz.

**Fig. 6:**
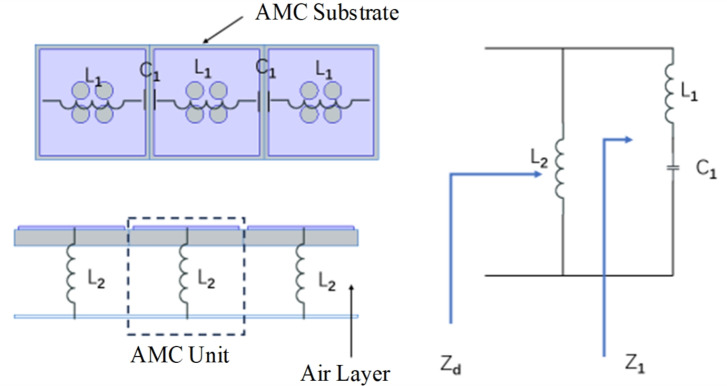
Equivalent circuit model of the AMC unit.

The center frequency of the AMC unit is determined by its equivalent LC resonant circuit. The AMC unit structure can be equivalent to a parallel LC resonant circuit using the equivalent circuit analysis method, whose equivalent circuit diagram is shown in Fig. 6. In the AMC unit structure, the gap between adjacent periodic structure patches can be equivalent to a capacitor $C_1$, each patch can be independently equivalent to an inductor $L_1$, and the air layer between the dielectric board and the floor can be equivalent to an inductor $L_2$. Therefore, the resonance frequency *f* and reflection coefficient $\Gamma$ of the proposed AMC can be expressed by Eqs. 2 and 3:

2 $$\begin{aligned} f= & \frac{1}{2\pi \sqrt{(L_{1} + L_{2})C_{1}}} \end{aligned}$$

3 $$\begin{aligned} \Gamma= & ln\Phi =\frac{Z_{S}-\eta _{0}}{Z_{S}+\eta _{0}} \end{aligned}$$

In Eq. 2, $L_1$ is mainly determined by the area of the metal patch and the height of the air layer, while $C_1$ is primarily determined by the gaps between the units. By adjusting the patch length *L* and the air layer height $H_2$, the operating frequency *f* and its corresponding $\pm 90^\circ$ phase bandwidth are aligned with the 3.1–4.1 GHz operating frequency band of the antenna.

In Eq. 3, $\Phi$ is the reflection phase. Then, the impedance bandwidth BW and the total surface impedance $Z_S$ of the AMC unit structure are respectively:

4 $$\begin{aligned} BW= & \frac{\pi }{8\eta _{0}}\sqrt{\frac{L_{1}+L_{2}}{C_{1}}\times (\frac{L_{2}}{L_{1}+L_{2}})^{2}} \end{aligned}$$

5 $$\begin{aligned} Z_{S}= & j2\pi fL_{2}\frac{1-(2\pi f)^{2}L_{1}C_{1}}{1-(2\pi f)^{2}(L_{1}+L_{2})C_{1}}=j\omega L_{2}\frac{1-\omega ^{2}L_{1}C_{1}}{1-\omega ^{2}(L_{1}+L_{2})C_{1}} \end{aligned}$$

At the parallel resonance frequency, the denominator of Eq. 5 is equal to zero. Therefore, the surface impedance $Z_S$ approaches infinity. The reflection coefficient of the AMC can be calculated by Eq. 3, where $\eta _0$ is equal to the impedance of air, then the reflection coefficient is 1 and the reflection phase is zero. Thus, the AMC unit structure proposed in this paper realizes zero-phase reflection.

**Fig. 7:**
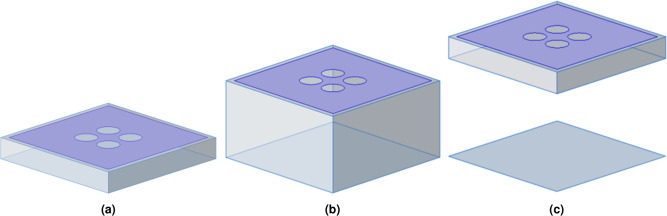
Design evolution of the AMC unit structure: (**a**) AMC 1, (**b**) AMC 2, (**c**) AMC 3.

**Fig. 8:**
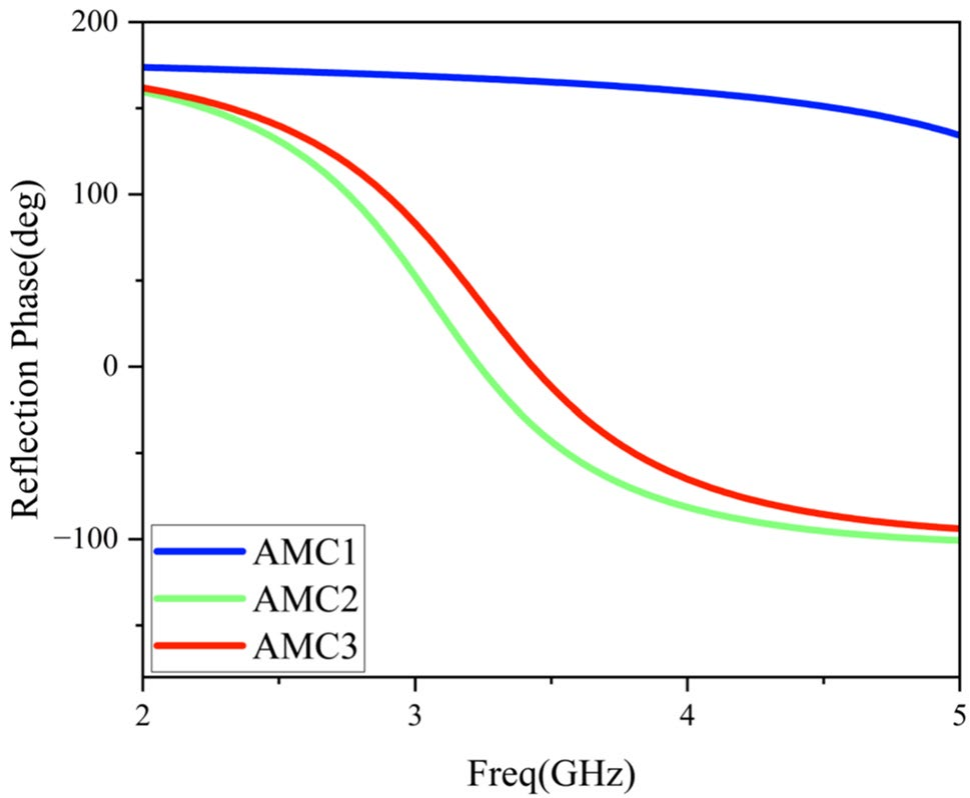
Influence of three AMC unit structures on reflection phase.

The design evolution of the AMC surface is shown in Fig. 7. Compared with AMC 1, the dielectric board of the AMC 2 structure is very thick; compared with AMC 1 and AMC 2, there is a certain air layer between the dielectric board and the floor of the AMC 3 structure. The simulation results of the three different structures are shown in Fig. 8. From the simulation results, it can be seen that increasing the thickness of the dielectric board will increase the bandwidth of the reflection phase of the AMC structure. However, this will increase the dielectric loss and production cost. Moreover, among the AMC structure units with the same overall height, the AMC 3 with the air layer structure has a higher reflection phase bandwidth. This is because the air layer can be equivalent to an inductor. Therefore, increasing the air layer will expand the reflection phase bandwidth of the AMC structure.

**Fig. 9:**
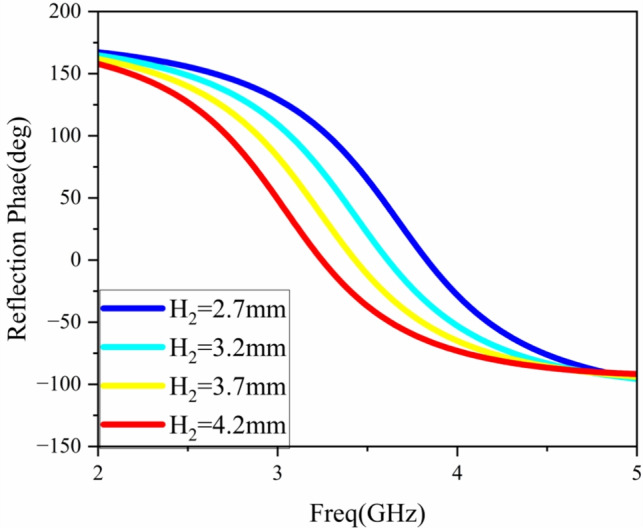
The reflection phase varies with the height $H_2$ of the air layer.

According to Eq. 4, the inductor $L_2$ is proportional to the bandwidth BW, that is, the height of the air layer is proportional to the bandwidth of the reflection phase. To determine the height of the air layer, Fig. 9 shows the influence of the height $H_2$ of the air layer on the reflection phase. It can be seen from the figure that the value range of $H_2$ is 2.7–4.2 mm. As the height of the air layer increases, the reflection phase of the AMC unit gradually increases. When $H_2$ is 4.2 mm, the reflection phase of the AMC unit reaches the widest. However, considering that the designed antenna is a low-profile base station antenna, that is, the overall height of the antenna should be as low as possible, so $H_2$ is selected to be 3.7 mm.

**Fig. 10:**
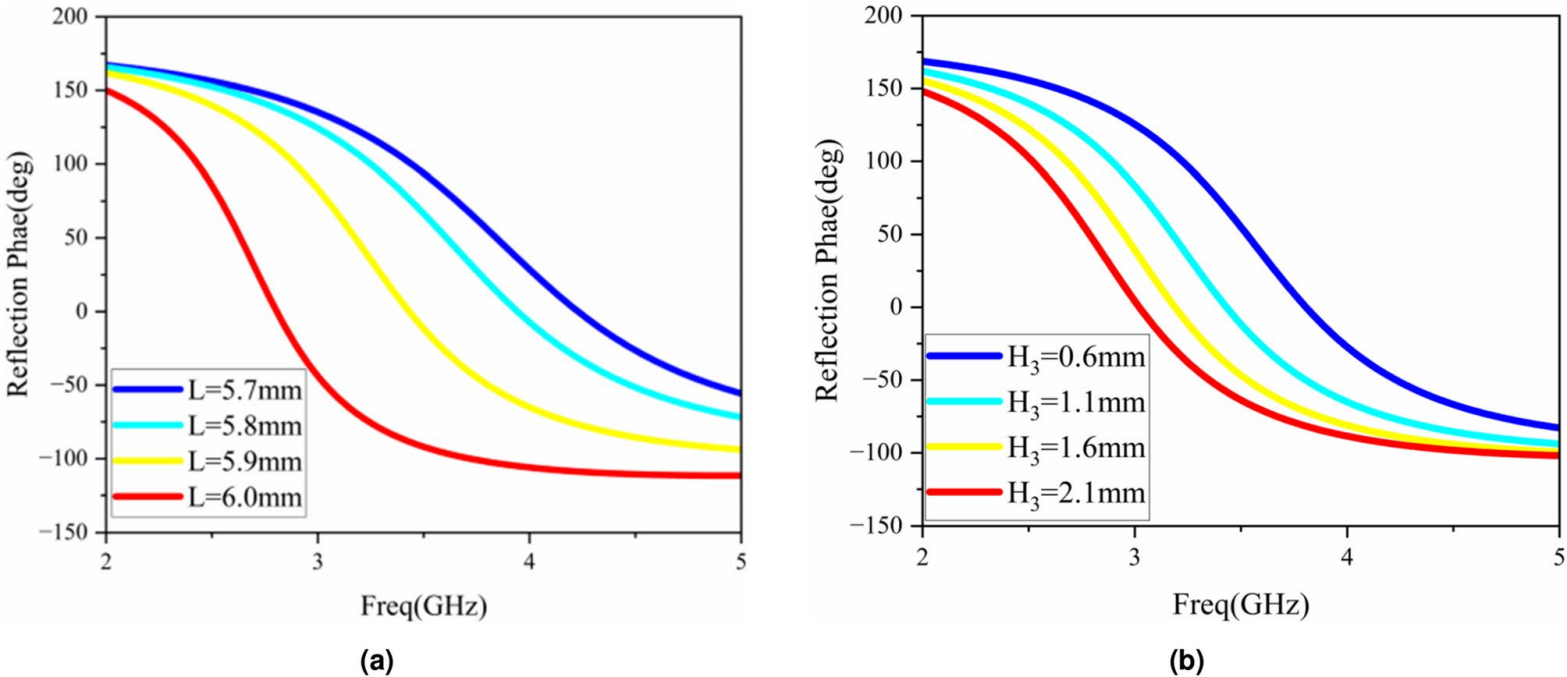
Results of the reflection phase varying with parameters: (**a**) patch length L, (**b**) thickness $H_3$ of the dielectric plate.

From the above formulas and Fig. 7, it is known that in addition to the height of the air layer and the thickness of the dielectric board, the factors affecting the performance of the AMC structure also include the length of the surface patch and the thickness of the dielectric board. Figure 10a shows the influence of the patch length L on the reflection phase bandwidth. From the equivalent circuit of the AMC, it can be seen that the patch is equivalent to an inductor. As the patch length L increases, that is, as the inductor component increases, the reflection phase bandwidth of the AMC structure unit shifts to the low frequency. The influence of the dielectric board thickness $H_3$ on the reflection phase of the AMC structure is shown in Fig. 10b . The thickness of the dielectric board is 0.6–2.1 mm. As the thickness of the dielectric board increases, the reflection phase bandwidth of the AMC structure gradually becomes wider. Considering the profile problem of the antenna, $H_3$ is selected to be 1.6 mm.

### Results and analysis

According to the design requirements of the antenna, the parameters of the designed low-profile antenna are analyzed and optimized through the electromagnetic simulation software HFSS. The optimal performance of the antenna is determined by adjusting the profile height of the antenna and the number of AMC structure units.

#### Simulation results and analysis

**Fig. 11:**
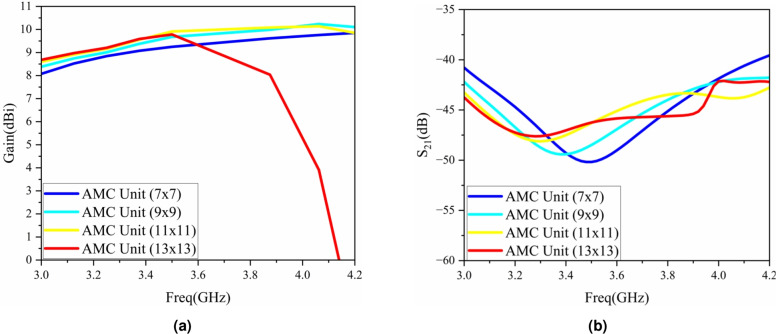
Influence of the number of AMC units on antenna performance: (**a**) gain, (**b**) isolation.

The number of different AMC structure units has an impact on both the port isolation and gain. As can be seen from the simulation results shown in Fig. 11, different numbers of AMC structure units cause irregular changes in the port isolation and gain. The designed antenna requires the isolation to be greater than 42 dB and the gain to be greater than 8.5 dBi. As can be seen from the figure 11, when the number of units is 7$\times$7, 9$\times$9, or 13$\times$13, the gain and isolation of the antenna are relatively low. Therefore, the number of AMC units is finally selected to be 11$\times$11.

**Fig. 12:**
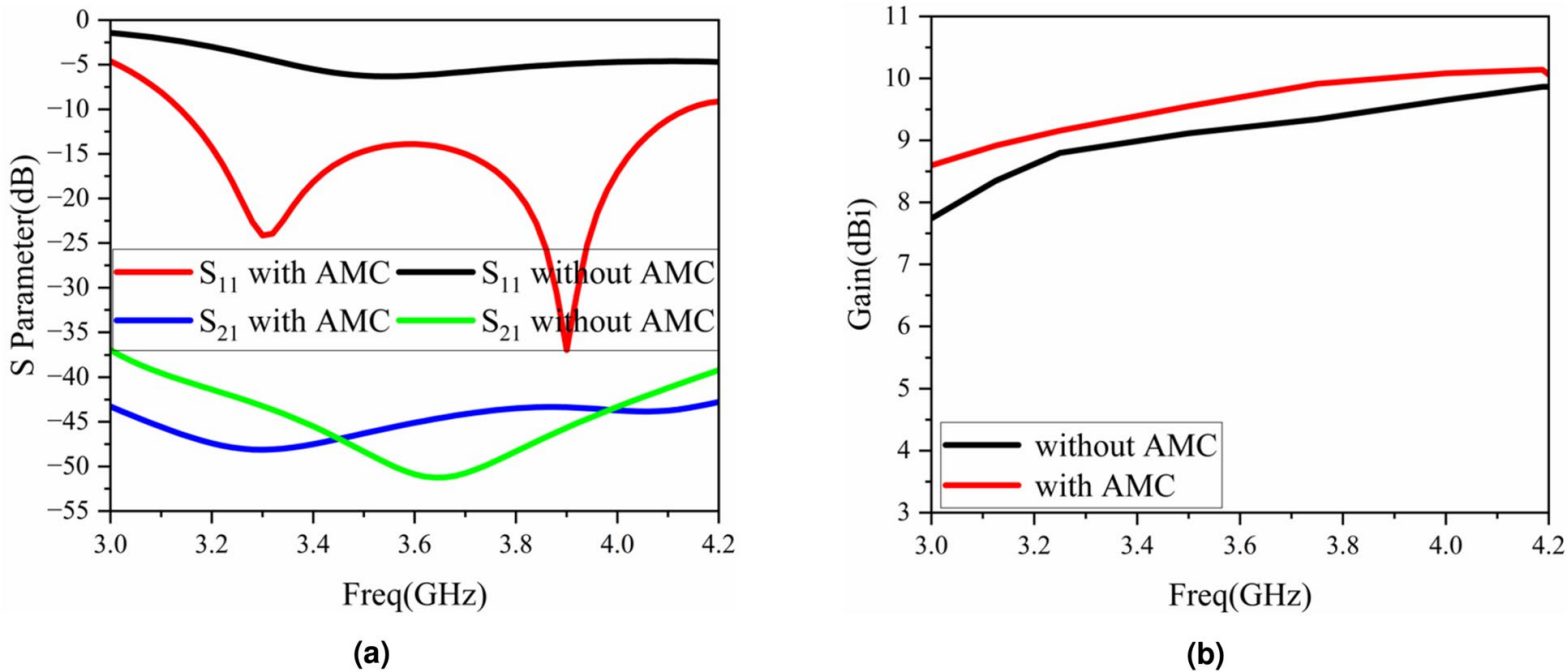
Comparison of S-parameters and gain for low-profile parameters: (**a**) S-parameters, (**b**) gain.

After loading the AMC surface on the antenna unit, the performance of the antenna has changed greatly. Figure 12 shows the comparison of the S-parameters and gain of two low-profile antennas, one with and the other without the loaded AMC. As can be seen from Fig. 12a , after loading the AMC surface, a new resonance point is introduced at high frequencies. The impedance bandwidth of the antenna is expanded through two resonance modes, and the impedance matching of the antenna is significantly improved. At the same time, loading the AMC surface has a certain coupling suppression effect on the two ports of the antenna. The isolation of the antenna without loading the AMC surface is greater than about 38 dB, while the isolation of the antenna after loading the AMC is greater than 42 dB within the operating bandwidth. Figure 12b is the comparison of the antenna gain. After loading the AMC surface, the gain of the antenna is greater than 8.5 dBi within the operating frequency band.

#### Measured results and analysis

**Fig. 13:**
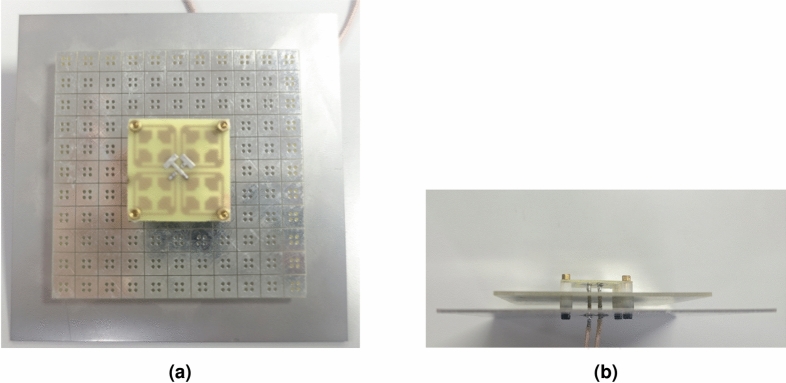
Fabricated antenna: (**a**) top view, (**b**) side view.

To verify the accuracy and practicality of the antenna design, the antenna is physically fabricated and tested according to the final parameters of the antenna. The antenna is shown in Fig. 13. At the same time, the port characteristics and radiation characteristics of the physical antenna are tested, and finally, a comparative analysis is carried out with the simulation results of the antenna.

**Fig. 14:**
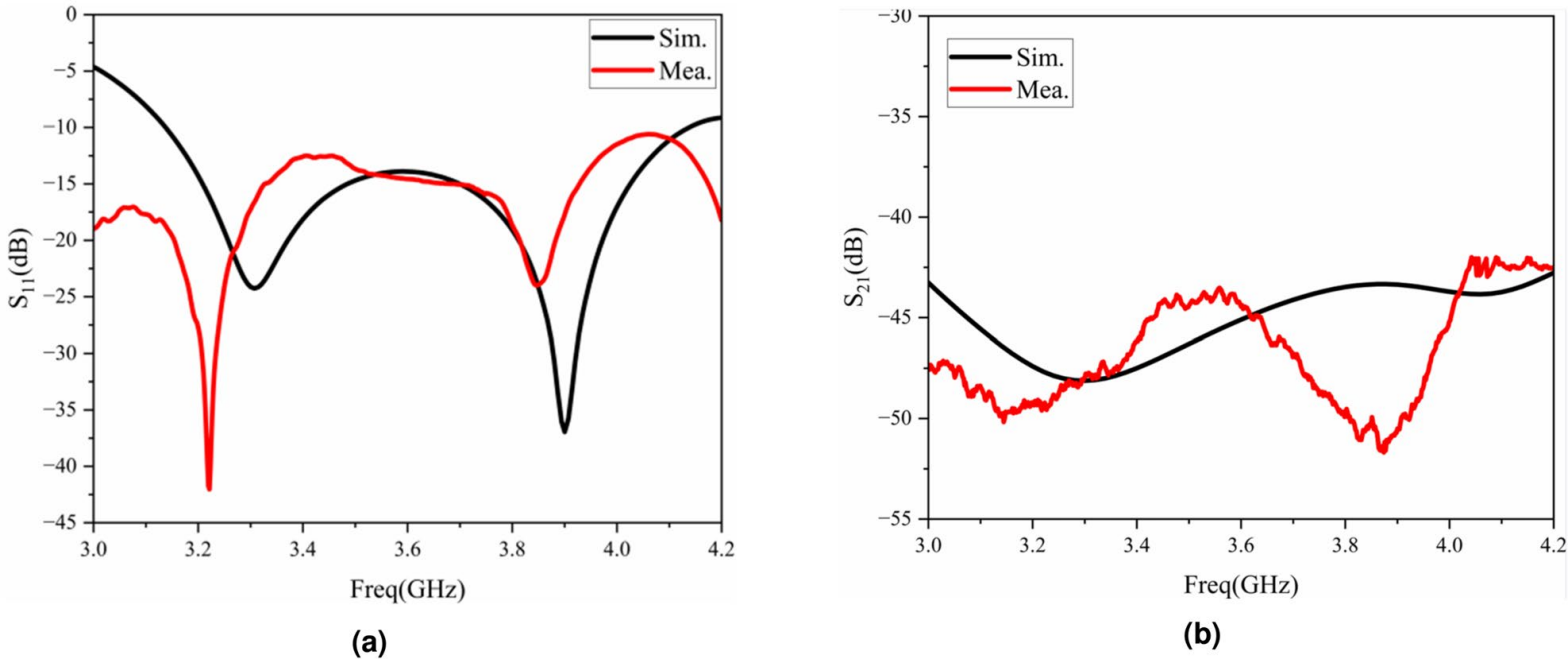
Comparison between the simulation and measured results of the antenna: (**a**) S-parameters, (**b**) voltage standing wave ratio.

Figure 14 shows the comparison diagram of the simulation and measured results of the S-parameters of the low-profile base station antenna. As can be seen from Fig. 14a , the $-10$ dB impedance bandwidth of the base station antenna is 3.1–4.1 GHz. The difference between the simulation and measured results mainly comes from the errors during the processing and soldering. Figure 14b shows the comparative analysis of the antenna isolation. It can be seen that there is a difference between the measured isolation of the antenna and the simulation. This is mainly because there are certain errors when the feeding part of the physical antenna is welded to the coaxial cable connector. However, within the operating frequency band, both the simulation and measured results of the isolation are greater than 42 dB, meeting the design specifications of the base station antenna.

**Fig. 15:**
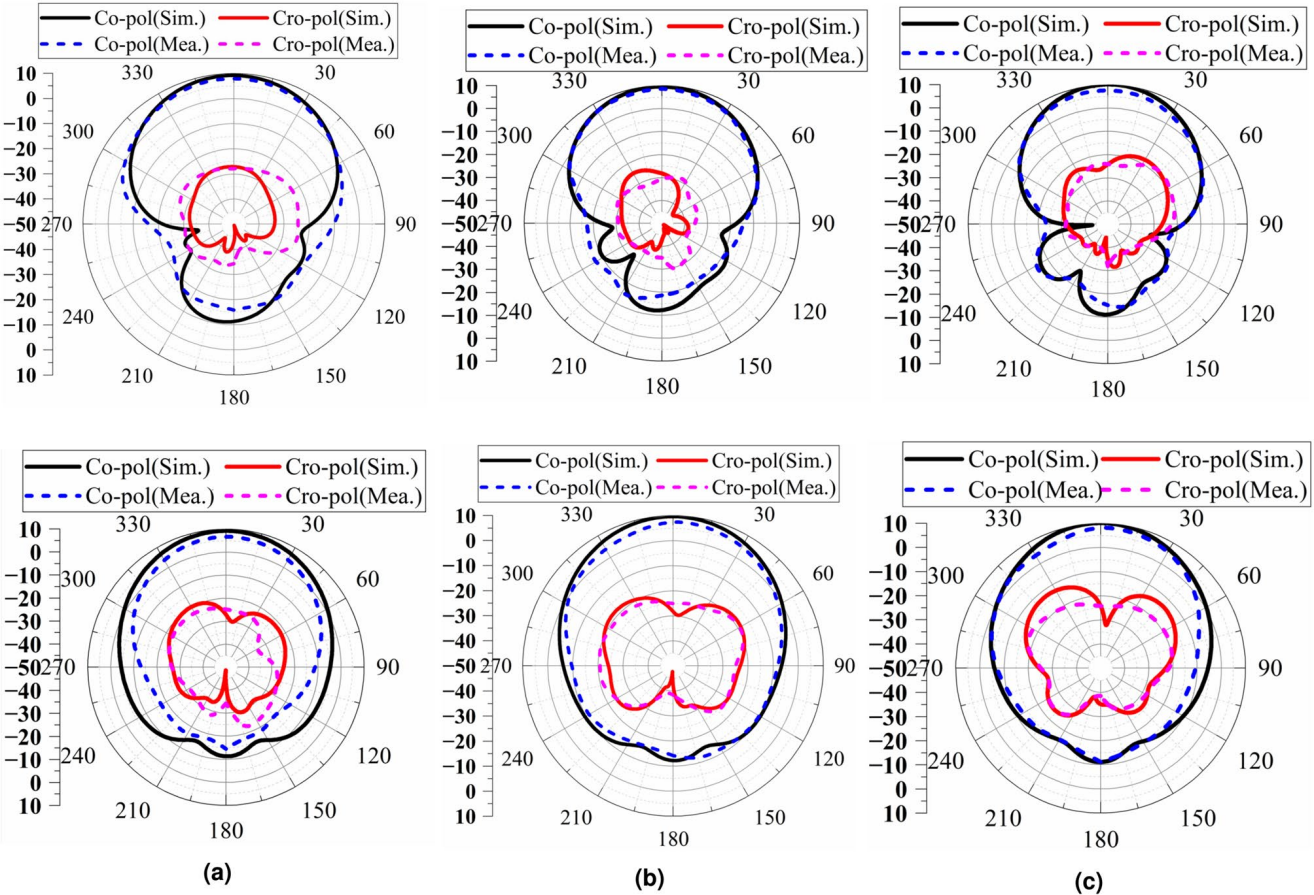
Simulation and measured radiation patterns of the low-profile antenna at 3.3 GHz (**a**), 3.6 GHz (**b**), and 3.9 GHz (**c**) in the E-plane (upper row) and H-plane (lower row).

Figure 15 shows the simulation and measured radiation patterns of the low-profile antenna at the frequency points of 3.3 GHz, 3.6 GHz, and 3.9 GHz in the E-plane and H-plane. Among them, co-pol represents the main polarization of the antenna, and cro-pol represents the cross-polarization of the antenna. From the simulation and test results, it can be seen that the cross-polarization of the antenna is greater than 22 dB, all meeting the design specifications of the radiation performance of the base station antenna. The measured results of the physical object also show that the antenna has better radiation performance.

#### Performance comparative analysis

Table 2 shows the comparison of the profile height and other performances of the low-profile base station antenna developed in this paper with those of typical international products^19–23^. Among them, $\lambda$ is the wavelength of the center frequency. It can be seen that the relative bandwidth of the antenna developed in this paper is basically the same as that of the international representative products, but it achieves better port isolation, effectively reducing the interference between channels, and maintaining a relatively high radiation gain. Finally, it is particularly outstanding in terms of the low-profile characteristics, and its profile height is significantly lower than that of most of the compared antennas. These advantageous features make the antenna design have important engineering application value in application scenarios such as 5G communication systems where there are strict restrictions on the antenna height.

**Table 2:** Comparison of antenna performance.

Work	Operating frequency (GHz)	impedance bandwidth(%)	cross polarization(dB)	Isolation (dB)	Gain (dBi)	Profile height (mm)
Ref.^19^	1.65-2.81	53.9	–	$>30$	$>8.0$	0.13$\lambda$
Ref.^20^	1.58-2.79	55.4	$>30$	$>30$	$>9.5$	0.13$\lambda$
Ref.^21^	1.67-2.98	58.7	$>20$	$>25$	$>8.0$	0.12$\lambda$
Ref.^22^	3.10-5.20	51.5	$>29$	$>30$	$>8.5$	0.12$\lambda$
Ref.^23^	1.69-3.81	77.0	–	$>27$	$>7.5$	0.15$\lambda$
This Work	3.10-4.10	27.8	$>22$	$>42$	$>8.5$	0.11$\lambda$

## Conclusion

This paper presents the development of a novel low-profile dual-polarized antenna designed for 5G Massive MIMO base station applications, achieving both miniaturization and high-performance characteristics. It holds significant importance for the integrated development of mobile communication base station arrays. By expanding the proposed artificial magnetic conductor (AMC) unit structure into an 11$\times$11 array reflector to replace the traditional metal ground plane, the antenna’s profile height is significantly reduced. Through simulation optimization and experimentation, the overall dimensions of the low-profile antenna are determined to be 0.28$\lambda$ $\times$ 0.28$\lambda$ $\times$ 0.11$\lambda$ (where $\lambda$ is the wavelength corresponding to the center frequency). To verify the accuracy and practicality of the antenna, a prototype was fabricated and tested. The results demonstrate outstanding performance within the operational frequency band of 3.1-4.1 GHz: Port isolation exceeds 42 dB, Gain surpasses 8.5 dBi. Furthermore, the antenna features a simple, compact structure that is easy to manufacture, providing new technical insights and solutions for the low-profile design of 5G Massive MIMO base station antennas.

## Data Availability

All data generated or analysed during this study are included in this published article.
